# Supplementary material for: Evaluating Fatalism Among Breast Cancer Survivors in a Heterogeneous Hispanic Population: A Cross-Sectional Study
Source: Curr Oncol. 2025 Aug 15;32(8):461. doi: 10.3390/curroncol32080461 (PMC12384765; doi:10.3390/curroncol32080461)
Supplement: Supplementary file 1 [file curroncol-32-00461-s001.zip › Supplemetary Figure S1.pdf]

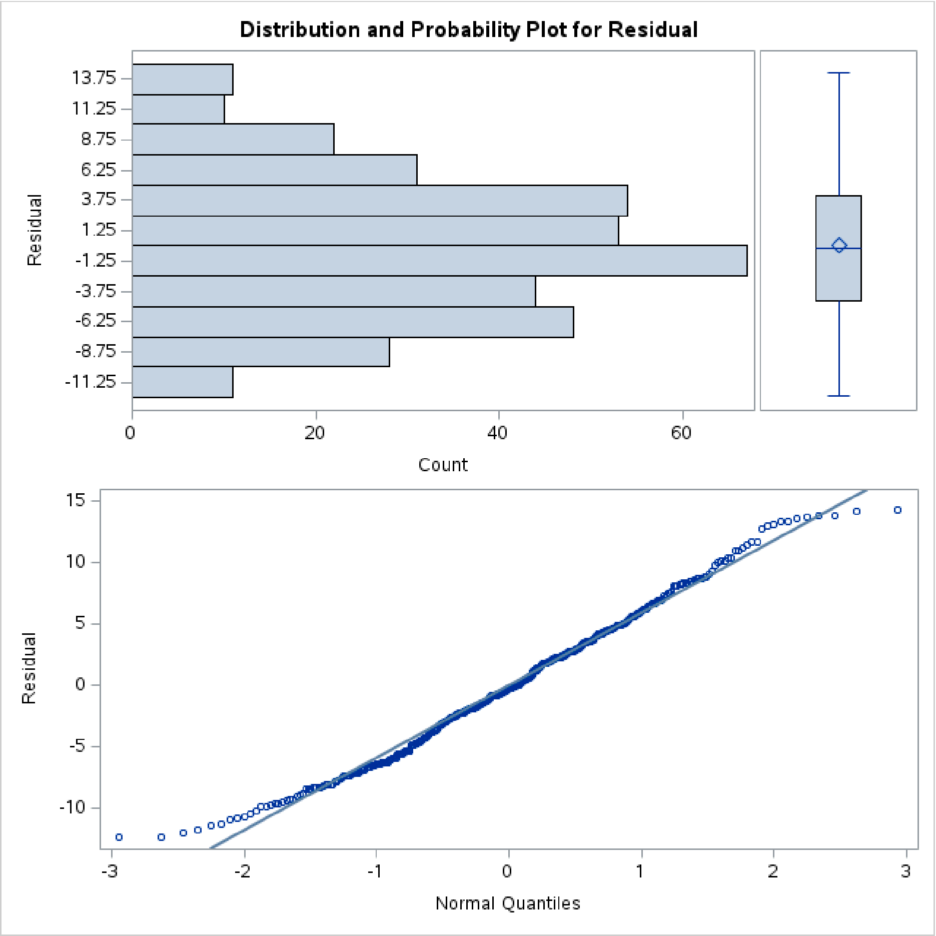

Supplementary Figure S1. A graphic examination for the residual of the multivariable linear model. The residuals appear to be symmetrically distributed with a bell-shaped pattern, suggesting approximate normality. Additionally, the Q-Q plot indicates that the residuals align closely with the theoretical quantiles of a normal distribution.
