# Supplementary material for: Evaluating Fatalism Among Breast Cancer Survivors in a Heterogeneous Hispanic Population: A Cross-Sectional Study
Source: Curr Oncol. 2025 Aug 15;32(8):461. doi: 10.3390/curroncol32080461 (PMC12384765; doi:10.3390/curroncol32080461)
Supplement: Supplementary file 1 [file curroncol-32-00461-s001.zip › Supplemetary Table S1.pdf]

Supplementary Table S1. A sensitivity analysis: Results from a multivariable linear regression model with complete cases (N= 379)

**Characteristics**	**Parameter**	**b**	**SE**	***P*-value**
	Intercept	19.3	2.4	<.0001
Hispanic Origin (Ref: Puerto Rican)	Colombian	-4.0	1.2	**0.001**
	Cuban	-0.9	1.4	0.527
	Dominican	-0.1	1.2	0.945
	Mexican	1.3	1.5	0.395
	Venezuelan	1.3	1.4	0.357
	Other Hispanic	-0.7	1.1	0.522
Current age (Ref: 20 - < 40)	40 - < 55	-2.0	1.7	0.252
	55 - < 70	-2.9	1.7	0.099
	70 +	-3.3	1.8	0.075
Race (ref: white)	Non-white	0.6	0.7	0.327
Household income (Ref: < $20,000)	$20,000 - < $75,000	-2.3	0.9	**0.011**
	≥ $75,000	-2.4	1.1	**0.033**
	Prefer not to answer	-1.3	1.1	0.259
Education (Ref: ≤ High school)	Some college +	-1.9	0.8	**0.012**
Language use at home (Ref: More Spanish)	Both equally	-0.2	0.9	0.815
	More English	-2.0	1.0	**0.042**
Years lived in US (ref: < 10)	10 - < 30	0.9	1.0	0.390
	30+	1.5	1.1	0.147
Years since diagnosis (Ref: < 2)	2 - < 5	1.9	1.1	0.086
	≥ 5	1.9	1.1	0.099
Fear of recurrence (Ref: Low)	Moderate	0.8	0.7	0.276
	High	1.7	1.1	0.126

b: regression coefficient, SE: standard error
